# Association between patient characteristics, demographics, and social determinants of health with treatment-refractory and complex-to-manage psoriatic arthritis

**DOI:** 10.1016/j.ero.2026.100239

**Published:** 2026-09-17

**Authors:** Shikha Singla, Fabian Proft, Andre L Ribeiro, Philip Mease, Christine Lindsay, M. Cameron Hay

**Affiliations:** 1 Department of Rheumatology, Medical College of Wisconsin, Milwaukee, WI, USA; 2 Department of Gastroenterology, Infectiology and Rheumatology (including Nutrition Medicine), Charité Universitätsmedizin Berlin, Berlin, Germany; 3 Department of Rheumatology, Hospital de Clínicas de Porto Alegre, Porto Alegre, Brazil; 4 Department of Rheumatology, Hospital Moinhos de Vento, Porto Alegre, Brazil; 5 Department of Rheumatology, Swedish Medical Center and University of Washington School of Medicine, Seattle, WA, USA; 6 GRAPPA Patient Research Partner, St. George, UT, USA; 7 Department of Anthropology, Miami University, Oxford, OH, USA

## Abstract

**Objectives:**

Psoriatic arthritis (PsA) is a heterogeneous disease that presents challenges in both diagnosis and treatment. To address the disease burden, the Group of Research and Association of Psoriasis and Psoriatic Arthritis conducted surveys with patients to gain insights into patient experiences regarding treatment-refractory (TR-) and complex-to-manage (C2M-) PsA. The objective of this study was to analyse patient responses to open-ended questions.

**Methods:**

A descriptive inductive coding approach was applied in Dedoose software [SocioCultural Research Consultants, LLC (SCRC)] to systematically analyse demographic variables in terms of relevant thematic codes for health-related quality of life, work, and financial concerns.

**Results:**

We found that TR/C2M-PsA narratives consistently highlighted themes of negative impact on health-related quality of life, work, and finances, and these effects were perceived with varying intensities among both genders, across age groups, languages, and disease manifestations. Social determinants of health, including education attainment, occupation and relationship status, also played an important role in influencing patient responses regarding TR/C2M-PsA.

**Conclusions:**

This study addresses an important gap in the literature regarding the impact of TR/C2M-PsA on patient experience. We observed a critical role of demographic factors and social determinants of health in the narrative themes of patients with high disease burden.

**WHAT IS ALREADY KNOWN ON THIS TOPIC**

Treatment-refractory and complex-to-manage psoriatic arthritis (TR/C2M-PsA) significantly impairs patients’ physical function and overall well-being, resulting in reduced quality of life, limited work productivity, and increased healthcare utilisation. Despite the well-recognised impact of the disease, the specific patient characteristics that predispose individuals to a higher disease burden remain insufficiently understood.

**WHAT THIS STUDY ADDS**

• Our results highlight that the profound impact of TR/C2M-PsA on health-related quality of life, work, and finances is perceived with different intensities among both genders, across age groups, languages, disease manifestations, as well as social determinants of health.

**HOW THIS STUDY MIGHT AFFECT RESEARCH, PRACTICE OR POLICY**

• By highlighting common patterns in patient narratives, this study provides a foundational understanding of patient experiences. These findings serve as a stepping stone, and further research is required to validate whether these descriptive themes can eventually inform personalised or patient-centred care.

Alt-text: Unlabelled box dummy alt text

## INTRODUCTION

Psoriatic arthritis (PsA) is an autoimmune disease characterised by inflammation of the musculoskeletal system associated with skin and nail psoriasis that can affect 1% to 3% of the world’s population [1,2]. The diverse nature of PsA poses a challenge in both diagnosis and treatment of the disease [3]. The involvement of joints and enthesis significantly impacts patients’ physical and psychological function, leading to a reduction in their quality of life (QoL) [[4], [5], [6]]. Work-related limitations and increased healthcare consumption further exacerbate negative effects on patients’ QoL generally [7] and are associated with a substantial financial strain [8].

To address the disease burden, recent efforts have been undertaken to integrate patient experiences to gain insights into various aspects of the management of this multifaceted disease [9]. Patient-reported outcomes have become meaningful end points in randomised control trials [[10], [11], [12]]. Additionally, international guidelines for managing PsA are being developed considering the patients’ perspective [13,14].

The Group of Research and Association of Psoriasis and Psoriatic Arthritis (GRAPPA) took the initiative to establish guidelines to define complex-to-manage (C2M-) and treatment-refractory (TR-; also referred to as difficult-to-treat) PsA. This endeavour was a multistep process that involved review of the existing literature followed by incorporation of both patients’ and health care providers’ perspective to understand what constitutes TR/C2M-PsA [[15], [16], [17]].

The objective of this study was to qualitatively analyse the open-ended questions in the survey to gain insight into the ways TR/C2M-PsA impacts health-related QoL, with a special focus on the work-related and financial challenges faced by the participating PsA patients. As the survey was distributed to patients prior to the GRAPPA definitions, these results represent broader challenges associated with PsA in general.

## METHODS

The ethics approval for this study was received from the independent ethics committee of the Charité Universitätsmedizin Berlin, Germany (EA1/225/23). The study methodology has been reported previously [17]. Briefly, an electronic survey was designed in English language by GRAPPA members in collaboration with patient research partners in July 2023. The survey was divided into 3 sections, including demographics, structured questions, and 4 open-ended questions, each with a 300-word limit. We have previously reported the results of the structured questions [17]. The 4 open-ended questions were: (i) How would you define difficult-to-treat PsA? (ii) How would you define complex-to-manage PsA? (iii) In what ways does the PsA affect your life that are difficult to treat/complex to manage? (iv) Is there anything else you would like to add? The questionnaire was then translated into 9 additional languages using the DeepL platform. Verification of each of the 9 drafts was conducted by 2 GRAPPA members per language who were native speakers of the respective language.

Participation in the survey was voluntary. Participants were not offered any financial incentive and were allowed to skip any question. Participants had the choice to withdraw from the survey at any step. Confidentiality was maintained by keeping the questionnaire anonymous.

The objective of this study was to provide insight into patients’ perspectives through qualitative analyses of the open-ended questions.

### Analysis of open-ended questions

Demographic variables associated with participants constituted the quantitative data used in the mixed-methods analysis; specifically, we explored patterns in participant responses based on gender, age, ethnicity, primary language, marital status, education, and work status.

For the qualitative data analysis, a single coder [MCH] coded the entire dataset, ensuring consistency in code application. A descriptive inductive coding approach was applied using Dedoose, a secure software for mixed-methods analysis. Informed by literature on what matters to patients with PsA [18], the coding researcher read through 20% of the total, creating a preliminary list of potential codes to ensure that the concerns or themes of a response would be adequately captured. The code list was then built, and each response to the open-ended questions was coded over a period of about 3 weeks. Typically, multiple codes were applied to each response. For example, one response was “I am experiencing a gross inability to enjoy life from walking into a restaurant to taking a stroll in a park. I cannot perform basic tasks. I am worried for my future and that emotional toll has been severe”. This was ascribed the following codes: QoL concerns, uncertainty, future planning/daily activities, emotional distress/mental health, frustration, and activities of daily living (ADL). When a response could not be wholly captured by an existing code, a new code was added, and all responses in the data were reread to see if the new code needed to be applied.

**Figure 1:**
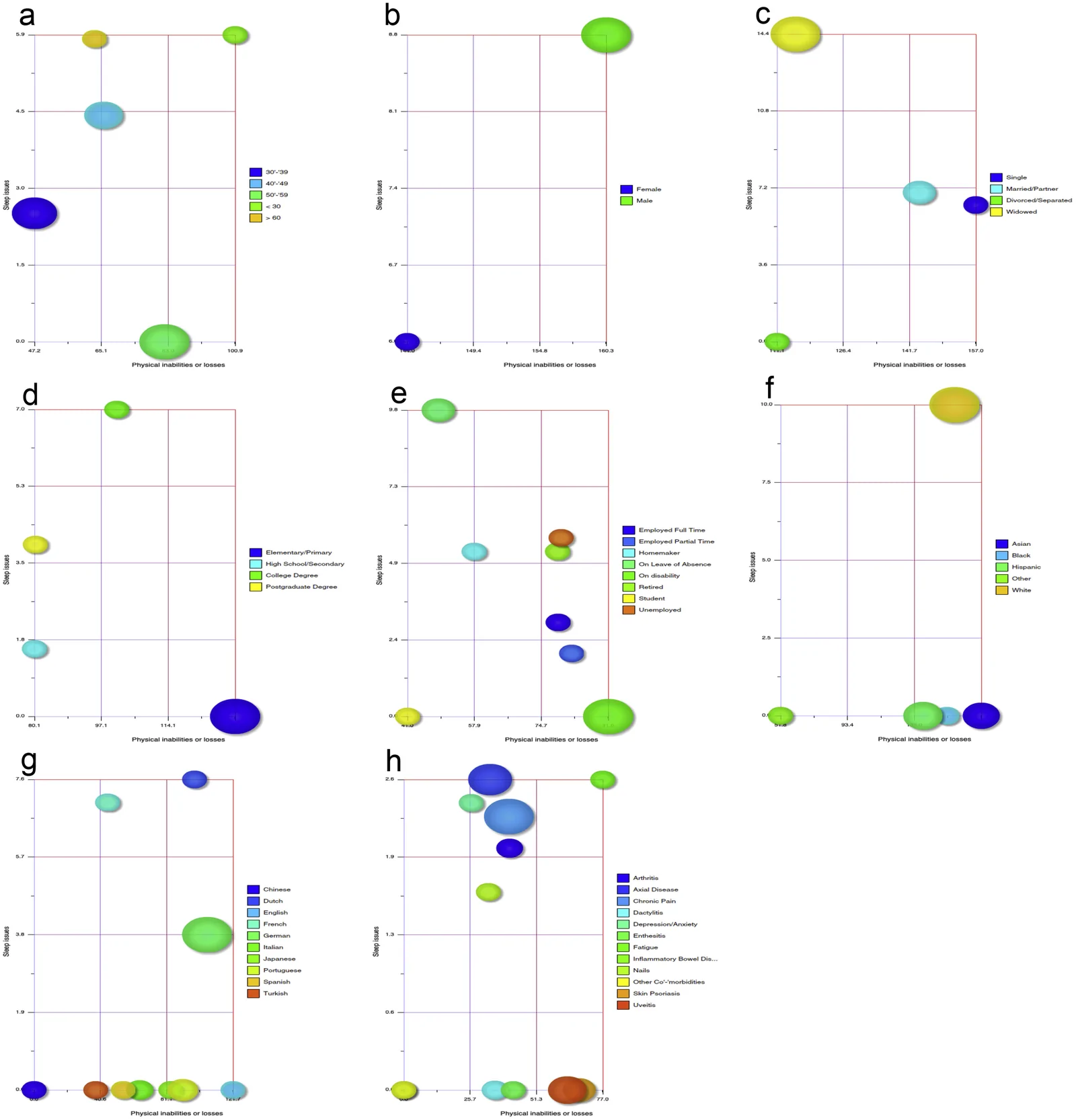
(a-h) Impact of treatment-refractory and complex-to-manage psoriatic arthritis on health-related quality of life.

**Figure 2:**
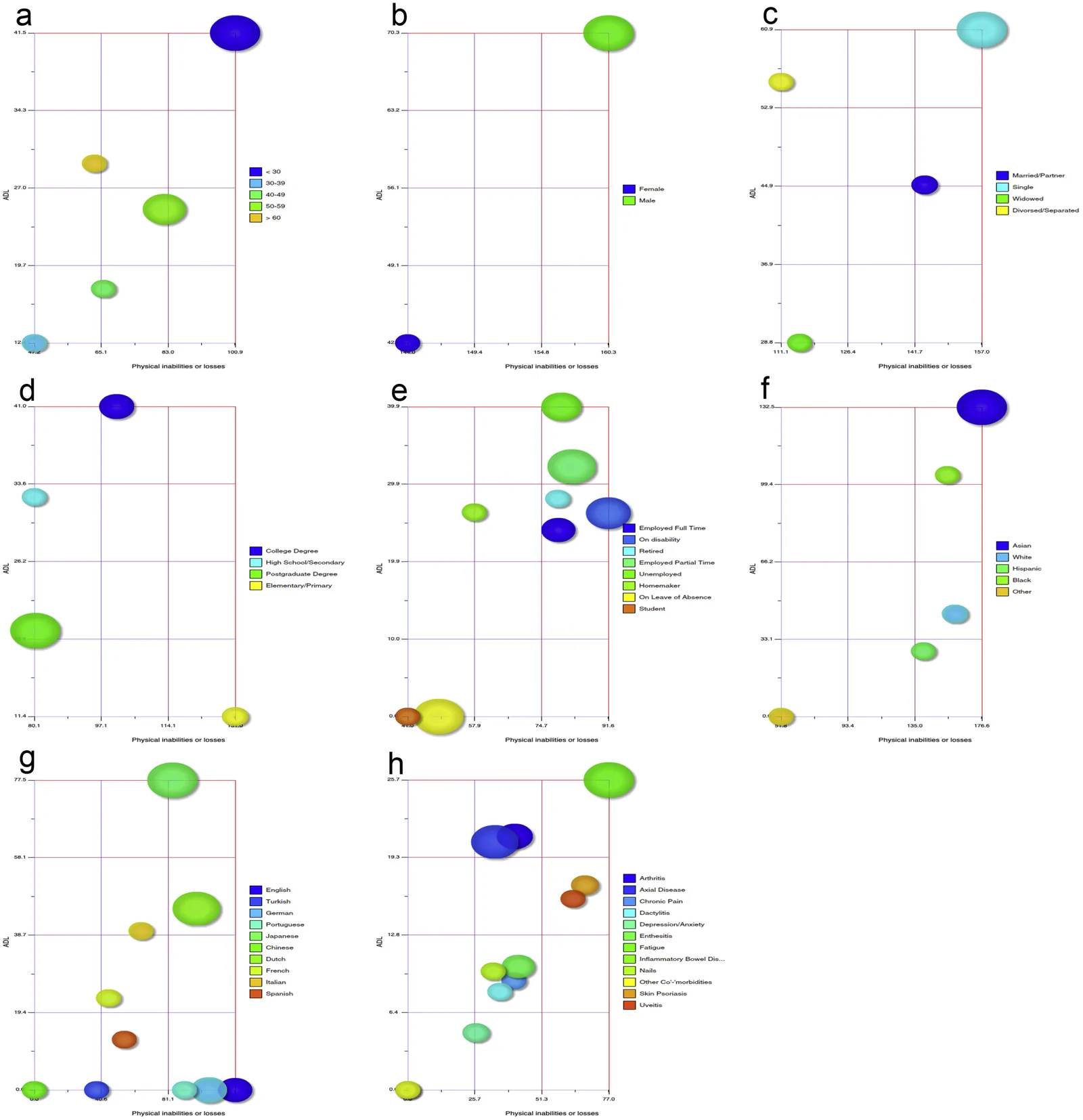
(a-h) Impact of treatment-refractory and complex-to-manage psoriatic arthritis on work. ADL, activities of daily living

**Figure 3:**
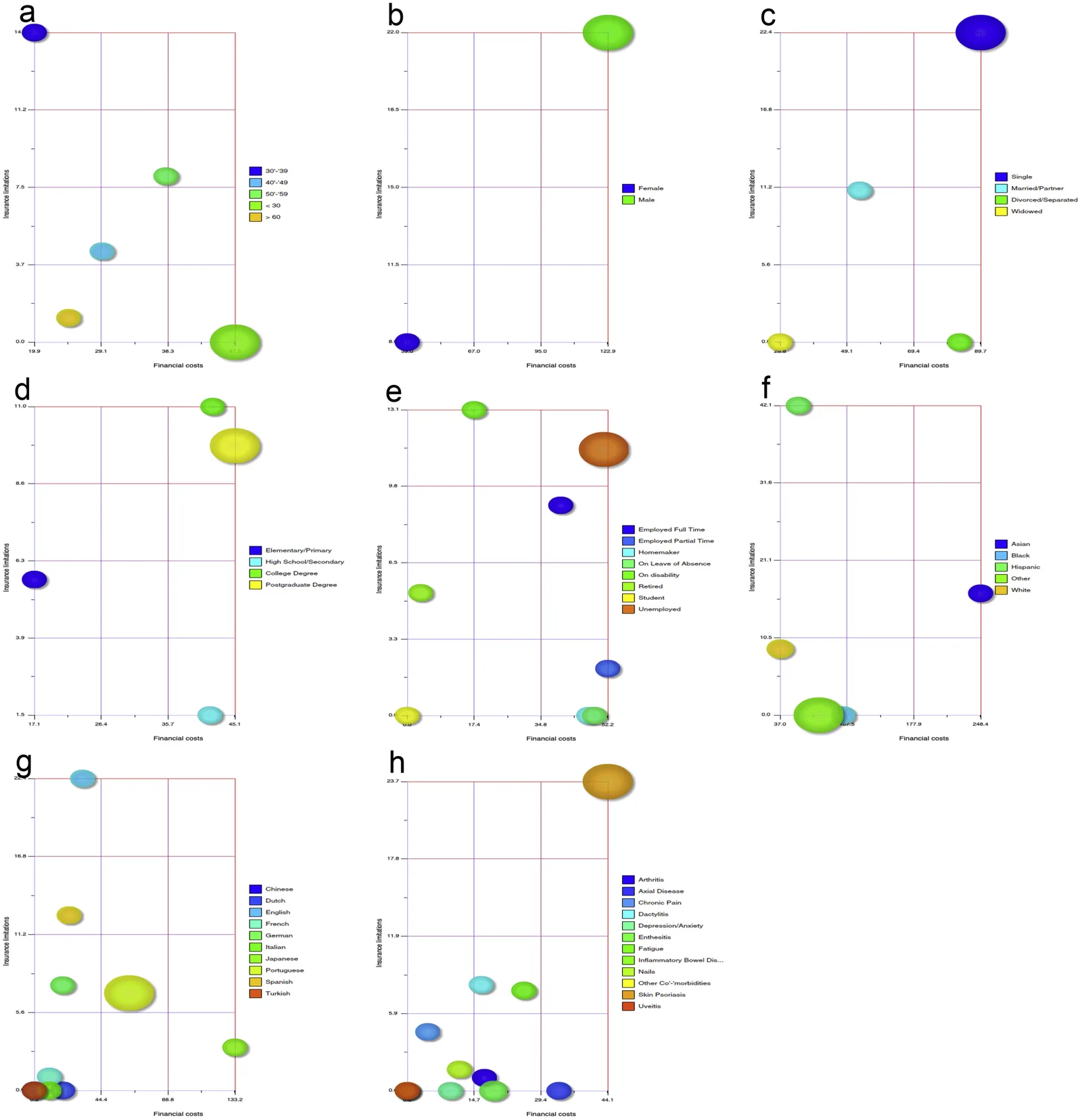
(a-h) Burden of treatment-refractory and complex-to-manage psoriatic arthritis on finances and time constraints.

Analysis in Dedoose affords thematic code co-occurrence based on the frequency with which qualitative responses contained 2 or more overlapping themes. Higher frequency indicates stronger associations between themes and how patients wrote about their experiences. Dedoose’s frequency bubble charts enable analysis of the relationships between 1 demographic or quantitative variable (eg, age or primary symptom) and 3 different thematic codes of the narrative responses. One of these codes is represented in the size of the bubble, and 2 are represented as code co-occurrence on the x- and y-axes (Figs 1-3).

To understand the ways TR/C2M-PsA impacts reports of health-related QoL, with a special focus on the work-related and financial challenges faced by the patients, we systematically examined each of the demographic variables against relevant thematic codes for health-related quality of life (HRQoL), Work, and financial concerns.

### Impact on health-related QoL

The size of the bubbles reflect the expanse of written comments that discussed PsA as a life-changing condition, with comments such as “It is a life-changing condition that does not allow any stability”, “An invisible thing that slowly eats away at you without you noticing it”, or “This condition controls my life in every way”. The y- and x-axes assessed remarks about sleep issues and physical incapabilities or losses, respectively.

Furthermore, a comparison was conducted to explore the relationships between emotional frustration, pain (which is both physical and psycho-emotional), and physical inabilities. The size of the bubble indicates the frustration implied in the participant’s writings, including comments like “It is frustrating to get your hopes up with a biological treatment, with all its adverse effects, and after 10 months they tell you that it did not work and the disease has jumped from stage 3 to 8 and you have to start again with another one with no guarantees” or “When new pain comes no one knows whether it's because of my main diagnosis [of] PsA or not and every time searching from beginning or like some doctors - without asking, say ‘that's your PsA, take your medicine’”. For this analysis, the y- and x-axes constitute the mentions of pain and physical inabilities or losses.

### Impact on work

To understand impact on work, the size of each bubble indicates the degree to which respondents discussed their work while answering the open-ended questions. Examples of comments coded as “work” include: “It’s harder on the joints which makes it hard to work”, “I’ve lost my job and everything is becoming difficult”, and “PsA affects my professional and social life because of persistent pain and chronic fatigue”. The y- and x-axes examined the relationship between comments regarding difficulties with ADL and physical inabilities or losses, respectively.

### PsA burden: financial and time constraints

To understand the financial and temporal burdens of TR/C2M-PsA, the size of each bubble indicates the participants remarked that disease management was time consuming. Examples of comments coded as “time consuming” include: “An unpredictable condition where the search for proper medication takes a very long time”, “Requiring use of numerous healthcare professionals in all areas affected by the disease, with an enormous amount of time to devote to its care”, and “I find it time-consuming and sometimes temporary, constant medication changes and progressive return of symptoms”. The y-axis represents comments about insurance limitations, and the x-axis refers to comments about financial concerns.

## RESULTS

### Patient population

The survey was completed by 570 participants. The maximum rate of response was received from French-speaking patients (33.1%), followed by Spanish (15.8%), Japanese (10.7%), and English (10%). The Table outlines the characteristics of the participants. Ethnicity was self-reported by the participants as White (72.6%), Asian (12.8%), Hispanic (10.3%), Black (1.4%), and Other (2.8%). Age distribution included 50 to 59 years (32.3%), ≥60 years (27.4%), and 40 to 49 years (22.1%). Notably, more women (68.8%) participated in the survey than men (31.2%).

**Table:** Characteristics of participants in treatment-refractory and complex-to-manage psoriatic arthritis survey

Characteristics	Number of respondents
Age group, n (%)	Total answers—570
Below 30 y	30 (5.2%)
Between 30-39 y	74 (12.9%)
Between 40-49 y	126 (22.1%)
Between 50-59 y	184 (32.3%)
Above 60 y	156 (27.4%)
Gender, n (%)	Total answers—570
Male	178 (31.2%)
Female	392 (68.7%)
Race and ethnicity, n (%)	Total answer—570
Asian	73 (12.8%)
Black	8 (1.4%)
Hispanic	59 (10.4%)
White	414 (72.6%)
Other	16 (2.8%)
Relationship status, n (%)	Total answers—570
Single	116 (20.3%)
Married/committed relationship	384 (65.6%)
Divorced/separated	54 (9.5%)
Widowed	26 (4.5%)
Highest level of education, n (%)	Total answers—570
Elementary school	43 (7.5%)
High school	159 (27.9%)
College	244 (42.8%)
Postgraduate	124 (21.7%)
Current occupational status, n (%)	Total answers—570
Homemaker	39 (6.8%)
Unemployed	36 (6.3%)
Student	5 (0.9%)
Employed partial time	102 (17.9%)
Employed full time	204 (35.8%)
On leave of absence	21 (3.7%)
On disability	47 (8.2%)
Retired	116 (20.3%)
Answers by language, n (%)	Total answers—570
French	189 (33.1%)
Spanish	90 (15.8%)
Japanese	61 (10.7%)
English	57 (10%)
Dutch	50 (8.7%)
German	50 (8.7%)
Italian	38 (6.7%)
Portuguese	27 (4.7%)
Turkish	5 (0.9%)
Chinese	3 (0.5%)
Diagnosis delay, mean (SD)	Total answers—570
Time to psoriasis diagnosis	5.1 y (9.5)
Time to psoriatic arthritis diagnosis	4.3 y (11.7)

Regarding social determinants of health (SDOH), 21.7% of participants reported that their highest level of education attainment was a postgraduate degree. This was followed by college (42.8%) graduates, high school (27.9%) graduates, and patients who had an elementary school (7.5%) level of education. Only about one-third of the patients were employed full time (35.8%). However, 17.9% reported that they were employed half time and 20.3% were retired. Relationship status was listed as married or committed by 65.6 %, followed by single, divorced/separated, and widowed (20.3%, 9.5%, and 4.5%, respectively). For the qualitative analysis, we focused on the open-ended questions, which were completed by 207 respondents, making a total of 293 comments. The bubble charts representing the results normalise the data based on the number of recipients to account for small sample sizes. Supplementary Table shows the frequency with which specific codes were applied to comments within each category. Notably, the coding density varies as a single respondent’s comments may have numerous codes applied to their open-ended response*.*

### Impact on health-related QoL

In total, 56 comments described PsA as a life-changing condition. Narrative themes of sleep issues and physical inabilities were most prominent in comments from younger (<30 y/o) men (Fig 1a,b). However, patients in the middle-age decades also frequently expressed that TR/C2M-PsA is a life-changing disease. Divorced or separated patients were relatively less likely to include themes of sleep and physical inabilities/losses in their comments (Fig 1c).

Interestingly, even though themes of sleep were absent in the text of elementary or primary education attainers and patients on disability, this group repeatedly mentioned the physically restrictive and life-changing nature of the disease (Fig 1d,e).

All ethnicities (except White) demonstrated lower prominence of concerns for sleep disturbance due to the disease and only 3 language groups (Germans, French, and Dutch) associated the lack of sleep with TR/C2M-PsA (Fig 1f,g).

Respondents who ranked chronic pain and axial PsA as the most concerning feature of TR/C2M-PsA also authored narratives framing that PsA was a life-changing disease (Fig 1h).

### Emotional components of pain

A total of 41 responses voiced frustration in thinking about TR/C2M-PsA. Narratives from younger (<30 y/o) respondents emphasised more on pain and physical inabilities, whereas the 30 to 39 y/o respondents voiced the most frustration, followed by the 50 to 59 y/o age group (Supplementary Fig S1a). Themes of frustration and pain appeared with greater frequency in comments from women than men, though both genders discussed pain and physical inabilities (Supplementary Fig S1b). Narratives from single respondents focused on all 3 variables, ie, frustration, pain, and physical inabilities (Supplementary Fig S1c).

While both college and elementary/primary educated participants represented frustration and pain in their written comments, the elementary educated participants and patients on disability underscored physical inabilities with greater frequency (Supplementary Fig S1d,e).

Asians discussed the themes of physical inabilities the most. (Supplementary Fig S1f). Turks, Dutch, and English narratives highlighted frustration, pain, and physical inabilities, respectively (Supplementary Fig S1g).

Themes of frustration were most prevalent in the text of respondents with troublesome skin psoriasis, followed closely by those with axial disease (Supplementary Fig S1h).

### Impact on work

In total, there were 148 responses that mentioned the theme of work. Narratives from young (<30-year-old), men, and single participants were considerably more likely to discuss work, ADL, physical inabilities, and losses (Fig 2a-c).

Regarding education attainment, postgraduate degree holders frequently commented on work, whereas narratives from those with an elementary and primary education level discussed physical inabilities and losses (Fig 2d). Figure 2e highlights the role of occupation. Comments from unemployed, part-time employed, and patients on disability or leave discussed the theme of work more frequently than those from the employed.

Narratives from Asian participants communicated the most about work, ADL, physical inabilities, and losses (Fig 2f). Language analysis further yielded that themes linking TR/C2M-PsA to work limitations were most prominent in the comments of Japanese and Dutch participants. Japanese speakers were also more likely to note themes of ADL restrictions, whereas English-speaking patients were more likely to discuss physical inabilities (Fig 2g).

Participants with fatigue and axial disease as their most pronounced aspect of TR/C2M-PsA documented themes of work limitations the most (Fig 2h).

### PsA burden: financial and time constraints

In total, 17 comments discussed the themes of time constraints or temporal concerns. Narratives from participants in their 30s (30-39 y/o) wrote most about insurance concerns, whereas the younger (<30 y/o) group underscored the time-consuming nature of TR/C2M-PsA and the cost associated with the disease (Fig 3a). Men and single patients most frequently discussed all 3 variables ie, time constraints, insurance and cost (Fig 3b,c).

Comparison of education attainment revealed that the 2 groups with the higher (college and high school/secondary) degree frequently commented on financial and insurance concerns of TR/C2M-PsA (Fig 3d). Figure 3e shows the prominence of themes based on occupation. Unemployed individuals wrote about the time-consuming nature and cost of the disease, whereas patients on disability recited about the themes of insurance limitations.

Ethnicity data yielded interesting results as well. Individuals who self-identified as “Other” mentioned most about the time-consuming nature of the disease. Comments from Hispanics discussed insurance limitations the most (Fig 3f). Japanese speakers most frequently discussed financial costs, English speakers discussed insurance concerns, whereas Portuguese speakers discussed all 3 issues (Fig 3g).

Patients with skin psoriasis as their most concerning manifestation of TR/C2M-PsA shared the most comments regarding the financial burden of their disease (Fig 3h).

## DISCUSSION

The results from this study highlight that TR and C2M disease have a profound impact on the overall well-being of patients with PsA. Negative health-related QoL, as assessed by the frequency of comments regarding sleep deprivation and physical limitations, as well as bio-psychiatric comparisons of pain and physical impairments, was expressed with varying intensities among both genders, across age groups, languages, and SDOH. Additionally, the impact on work, measured by difficulties with daily activities and physical limitations, and the economic impact, assessed through insurance limitations and financial concerns, also varied in narrative prominence among different patient demographics, patient characteristics, and SDOH.

The variability in key impacts across sex suggests that men may be more likely to foreground concerns about difficulties at work, financial concerns, sleep difficulties, and losses in terms of physical abilities due to PsA, whereas females may foreground concerns about fatigue and pain. Multiple studies have noted variability in disease outcomes between men and women, to which our study contributes [19,20]. Eder et al [21] are currently conducting a large, international study to investigate whether and to what extent sex influences the onset and severity of PsA. This study will help to establish a foundation upon which we would be able to interpret the variability in disease perception between men and women.

Our study also underscores the global variability in how individuals articulate the PsA experience. The most difficult disease manifestations, as well as the patient-reported concerns associated with TR/C2M-PsA, varied in patterned ways by language. Japanese speakers discussed more about ADL restrictions and financial costs, whereas English-speaking patients were likely to focus on physical inabilities and losses as well as insurance issues. This variability in key impacts across language groups is particularly interesting because it implies 1 or more of 3 possible explanations. The first possibility is that PsA may tend to manifest itself differently in different contexts in response to different environmental factors that impact bodily immune systems, including diet, air, and water quality, and societal stress levels [22]. The second possibility is that the diagnosis and treatment of PsA may differ in different medical systems due to access to resources, resulting in different outcomes or concerns for patients [23]. Indeed, the variability in terms of patient concerns about the financial burdens of the disease that emerged in our data demonstrates that the health system design of country (private payer, public, or mixed) significantly impacts the financial burdens of patients. The third possibility is that the cultural norms for social roles, salient embodied sensations, and how experiences can be expressed can vary significantly in different cultural settings, as has been found in other studies [24,25]. Our study design does not allow these overlapping cultural and systemic factors such as socioeconomic status and geographic access to care to be definitively uncoupled, requiring future targeted research to evaluate these possibilities.

The role of individual and interpersonal SDOH, such as younger age, education attainment, occupation, and relationship status, has not been well studied in PsA. Our findings suggest that patients who are not in a relationship and those with elementary/primary education may have greater needs for support than other groups. Previous reports have also shown an association between low level of education with work impairment and greater financial cost of the illness [26].

Finally, our analyses indicate that patients with axial and cutaneous symptoms may necessitate more attention, as also observed in a study by Mease et al [27]. Each of these possibilities and their potential overlaps are intriguing and need systematic investigation to fully understand and characterise global variability in patient experience with TR/C2M-PsA.

Our findings address a critical gap in the literature regarding patient-centred care by identifying patient demographics and SDOH that most influence the thematic focus of experiences in TR/C2M-PsA.

The main strength of the study is that we have captured narrative data about human experiences given the qualitative nature of our analyses. We have examined intricately interconnected issues like work incapacity, financial constraints, and QoL concerns, in 10 diverse languages, representing a global perspective on TR/C2M-PsA.

A limitation of the study is that these were nonrandom samples of respondents from around the world. It is possible that the results could have been different if the target population had been randomly selected; however, this type of selection bias is difficult to confirm or control in survey-based research. Another limitation of survey-based research is the potential for recall bias and survey bias leading to tendencies to underscore problems in responses. Not all participants contributed to the open-ended questions and responders were not compared with nonresponders. Therefore, it is possible that the study may have overestimated the disease burden, as individuals with more severe conditions are more prone to submitting detailed narrative feedback. Furthermore, small sample sizes and uneven baseline characteristics across certain subgroups—most notably Turkish, Chinese, and Black cohorts—limit the generalisability of group-specific trends. Therefore, these findings represent localised narrative prominence rather than definitive cross-cultural differences. Finally, the disease activity data were not collected. Without precise disease severity data, it remains plausible that many of the observed variations stem from chronic systemic drivers such as long-term structural damage, nociplastic pain, or overlapping comorbidities and psychiatric conditions.

However, the survey was distributed by physicians who intended to understand patients’ perspective about TR/C2M-PsA.

In conclusion, our study highlights the varying narrative prominence of patient characteristics, demographics, and SDOH on TR/C2M-PsA experiences. Future research that differentiates the needs of people with PsA based on their primary complicating symptom as well as SDOH could provide valuable insights for patient-centred care.

## CRediT authorship contribution statement

**Shikha Singla:** Writing – review & editing, Writing – original draft, Methodology, Investigation, Data curation, Conceptualization. **Fabian Proft:** Writing – review & editing, Validation, Formal analysis, Conceptualization. **Andre L Ribeiro:** Writing – review & editing, Data curation, Conceptualization. **Philip Mease:** Writing – review & editing, Validation, Supervision, Conceptualization. **Christine Lindsay:** Writing – review & editing. **M. Cameron Hay:** Writing – review & editing, Methodology, Formal analysis, Data curation, Conceptualization.

## Appendix Supplementary materials

Image, application 1

Image, application 2

## Footnotes

Supplementary material associated with this article can be found in the online version at doi:10.1016/j.ero.2026.100239.
