# Supplementary material for: Association between patient characteristics, demographics, and social determinants of health with treatment-refractory and complex-to-manage psoriatic arthritis
Source: EULAR Rheumatol Open. 2026 Sep 17;2(4):100239. doi: 10.1016/j.ero.2026.100239 (PMC13601050; doi:10.1016/j.ero.2026.100239)
Supplement: Supplementary file 1 [file mmc1.pdf]

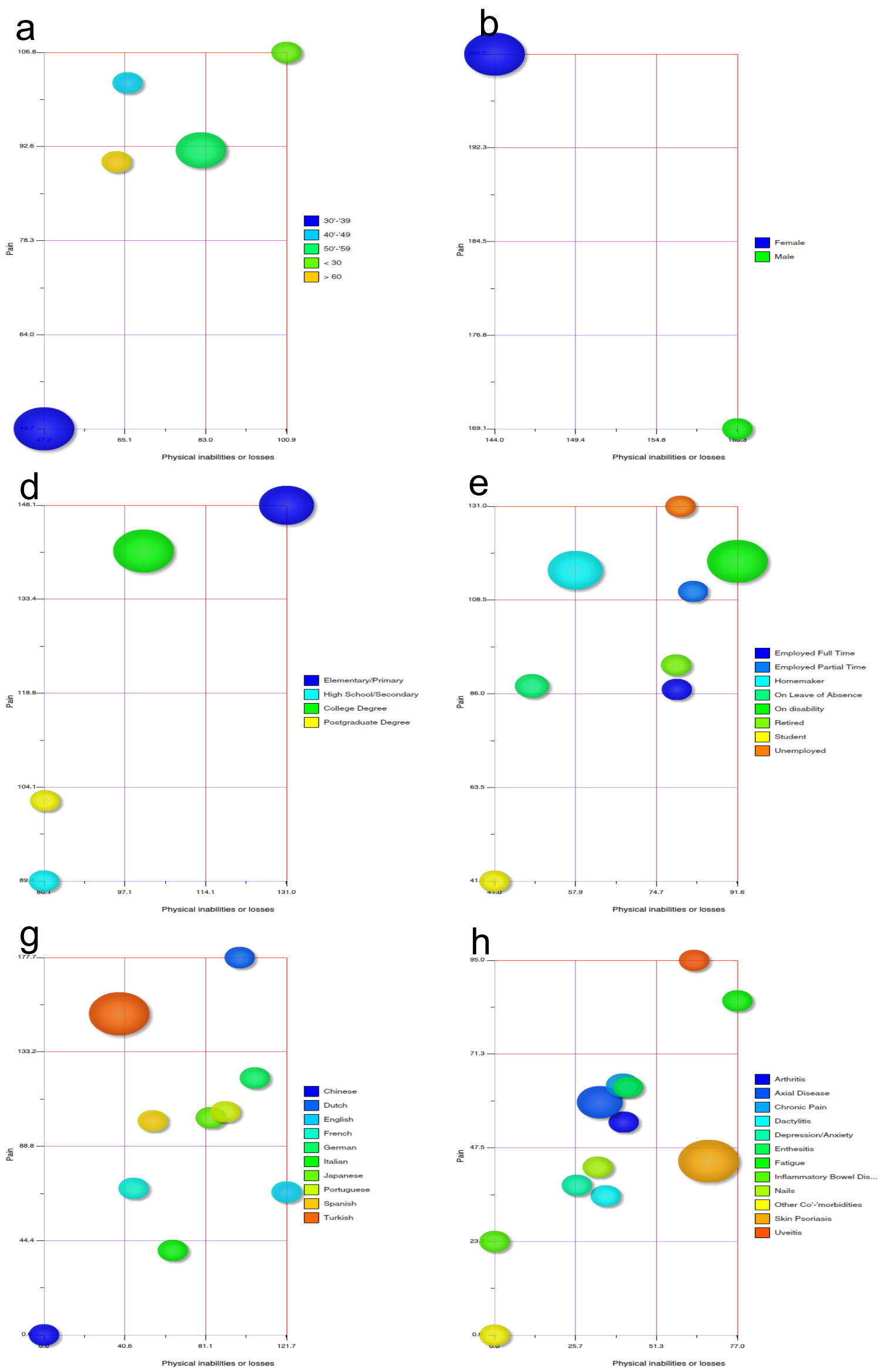
a
106.8
92.6
78.3
64.0
Pain
65.1
83.0
100.9
Physical inabilities or losses
30'-'39
40'-'49
50'-'59
< 30
> 60
b
192.3
184.5
176.8
169.1
Pain
144.0
149.4
154.8
Physical inabilities or losses
Female
Male
c
198.7
183.8
168.8
153.8
Pain
126.4
141.7
157.0
Physical inabilities or losses
Single
Married/Partner
Divorced/Separated
Widowed
d
148.1
133.4
118.8
104.1
Pain
97.1
114.1
131.0
Physical inabilities or losses
Elementary/Primary
High School/Secondary
College Degree
Postgraduate Degree
e
131.0
108.5
86.0
63.5
Pain
57.9
74.7
91.6
Physical inabilities or losses
Employed Full Time
Employed Partial Time
Homemaker
On Leave of Absence
On disability
Retired
Student
Unemployed
f
252.6
228.3
203.9
155.3
Pain
51.8
93.4
135.0
176.6
Physical inabilities or losses
Asian
Black
Hispanic
Other
White
g
177.7
133.2
88.8
44.4
Pain
40.6
81.1
121.7
Physical inabilities or losses
Chinese
Dutch
English
French
German
Italian
Japanese
Portuguese
Spanish
Turkish
h
95.0
71.3
47.5
Pain
25.7
51.3
77.0
Physical inabilities or losses
Arthritis
Axial Disease
Chronic Pain
Dactylitis
Depression/Anxiety
Enthesitis
Fatigue
Inflammatory Bowel Dis...
Nails
Other Co'-'morbidities
Skin Psoriasis
Uveitis
