# Supplementary material for: Association between patient characteristics, demographics, and social determinants of health with treatment-refractory and complex-to-manage psoriatic arthritis
Source: EULAR Rheumatol Open. 2026 Sep 17;2(4):100239. doi: 10.1016/j.ero.2026.100239 (PMC13601050; doi:10.1016/j.ero.2026.100239)
Supplement: Supplementary file 2 [file mmc2.pdf]

Category	Life changing condition	Sleep issues	Physical inabilities or losses	Frustration	Pain	Work	Activities of daily living	Time consumption	Insurance limitations	Financial costs
**Age**										
< 30	2	1	17	2	18	12	7	4	0	8
30-39	9	1	19	7	20	16	5	2	6	8
40-49	14	3	45	6	70	25	12	2	3	20
50-59	24	0	82	16	92	66	25	7	8	38
> 60	7	5	54	10	77	29	25	2	1	21
**Gender**										
Female	35	6	144	30	200	91	42	11	8	39
Male	21	4	73	11	77	57	32	6	10	56
**Relationship Status**										
Married/Partner	38	7	144	27	183	92	45	10	11	53
Single	11	2	49	11	62	42	19	7	7	28
Widowed	3	1	8	2	12	3	2	0	0	2
Divorsed/Separated	4	0	16	1	20	11	8	0	0	12
**Education**										
College Degree	21	7	101	24	141	67	41	4	11	42
High School/Secondary	11	1	52	7	58	32	21	4	1	27
Postgraduate Degree	15	2	41	6	52	38	10	8	5	23
Elementary/Primary	9	0	23	4	26	11	2	1	1	3
**Occupation**										
Employed Full Time	18	3	79	12	87	53	24	7	9	40
On disability	10	0	21	8	27	16	6	0	3	4
Retired	11	3	45	6	53	15	16	2	3	2
Employed Partial Time	9	1	41	4	55	38	16	3	1	26
Unemployed	2	1	14	3	23	11	7	4	2	9
Homemaker	3	1	11	6	22	7	5	1	0	9
On Leave of Absence	3	1	5	2	9	8	0	0	0	5
Student	0	0	1	0	1	0	0	0	0	0
**Ethnicity**										
Asian	6	0	32	9	36	35	24	1	3	45
White	45	10	160	30	195	102	44	14	9	37
Hispanic	5	0	20	2	36	9	4	1	6	8

Black	0	0	3	0	3	0	2	0	0	2
Other	0	0	2	0	7	2	0	1	0	3
**Language**										
English	4	0	38	16	21	21	0	0	7	10
Turkish	0	0	1	3	4	0	0	0	0	0
German	12	1	28	6	32	19	0	3	2	5
Portuguese	4	0	13	3	15	6	0	5	1	9
Japanese	5	0	27	3	33	32	25	1	1	43
Chinese	0	0	0	0	0	0	0	0	0	0
Dutch	6	2	26	2	47	25	12	1	0	5
French	14	7	45	6	69	31	23	5	1	10
Italian	5	0	13	1	8	6	8	0	0	2
Spanish	6	0	26	1	48	8	6	2	6	11
